# Differences in Clinician Electronic Health Record Use Across Adult and Pediatric Primary Care Specialties

**DOI:** 10.1001/jamanetworkopen.2021.16375

**Published:** 2021-07-09

**Authors:** Lisa S. Rotenstein, A. Jay Holmgren, N. Lance Downing, Christopher A. Longhurst, David W. Bates

**Affiliations:** 1 Brigham and Women’s Hospital, Boston, Massachusetts; 2 Harvard Medical School, Boston, Massachusetts; 3 Now with University of California, San Francisco; 4 Center for Biomedical Informatics Research, Department of Medicine, Stanford University School of Medicine, Stanford, California; 5 University of California at San Diego, La Jolla; 6 Harvard Chan School of Public Health, Boston, Massachusetts

## Abstract

This cross-sectional study examines differences in electronic health record use across adult and pediatric primary care specialties.

## Introduction

Clinicians spend a large proportion of their days using the electronic health record (EHR). There are known negative associations between measures of EHR use and clinician experience.^1^ Primary care clinicians spend significantly more total and after-hours time in the EHR than medical specialty and surgical colleagues.^2^ However, little is known regarding variation in EHR use across primary care specialties, such as between adult and pediatric care. In this cross-sectional study, we compared EHR use across general pediatrics, general internal medicine, and family medicine clinicians.

## Methods

The Stanford University institutional review board deemed this cross-sectional study exempt from approval and informed consent because it used deidentified data and was not considered human participant research. This report followed the Strengthening the Reporting of Observational Studies in Epidemiology (STROBE) reporting guideline.

The sample included 349 US-based ambulatory health care organizations using the same EHR vendor (Epic Systems) between January and August 2019. The sample included all clinicians with scheduled outpatient appointments, including physicians and advance practice practitioners.

Using EHR metadata,^3^ we measured total daily time actively using the EHR (time performing active tasks) and time spent after-hours per clinician (eMethods in the Supplement). EHR time was categorized into clinical review, notes, in-basket messages, and orders. We measured the mean number of messages received per clinician per day, overall and by source. We compared these metrics across general pediatric, general internal medicine, and family medicine clinicians. We used ordinary least squares regression to examine associations of total EHR time and after-hours EHR time with specialty, adjusting for organizational characteristics and mean daily patient volume per clinician, with robust SEs clustered at the organizational level. All analyses were conducted in Stata statistical software version 16.1 (StataCorp), with 2-sided α assessed at .05. Data were analyzed from January to March 2021.

## Results

**Figure 1.**
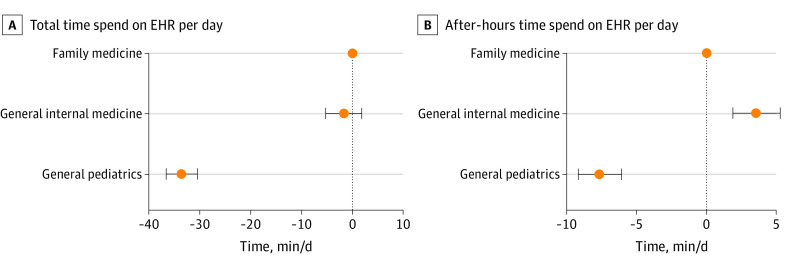
Comparisons of Total and After-Hours Time Spent on Electronic Health Records (EHR) Per Day Among Primary Care Specialty Types Point estimates and 95% CIs calculated using ordinary least squares regressions that include controls for organization type, organization structure, number of employed physicians, number of annual visits, and mean daily patient volume per clinician, with robust SEs clustered at the organization level.

A total of 349 health systems were included in the analysis. Clinicians across these organizations had a mean (SD) of 12.9 (4.4) encounters per day among general pediatrics clinicians, 11.5 (3.6) encounters per day among general internal medicine clinicians, and 12.8 (3.4) encounters per day among family medicine clinicians. Mean (SD) daily total active EHR time was 94.7 (26.3) minutes among general pediatrics clinicians, 121.5 (34.4) minutes among general internal medicine clinicians, and 127.8 (26.6) minutes among family medicine clinicians. Mean (SD) daily after-hours active time was 23.6 (11.0) minutes among general pediatrics clinicians, 34.4 (13.8) minutes among general internal medicine clinicians, and 31.2 (10.5) minutes among family medicine clinicians. Differences in total and after-hours time persisted in multivariable regression (Figure 1).

**Figure 2.**
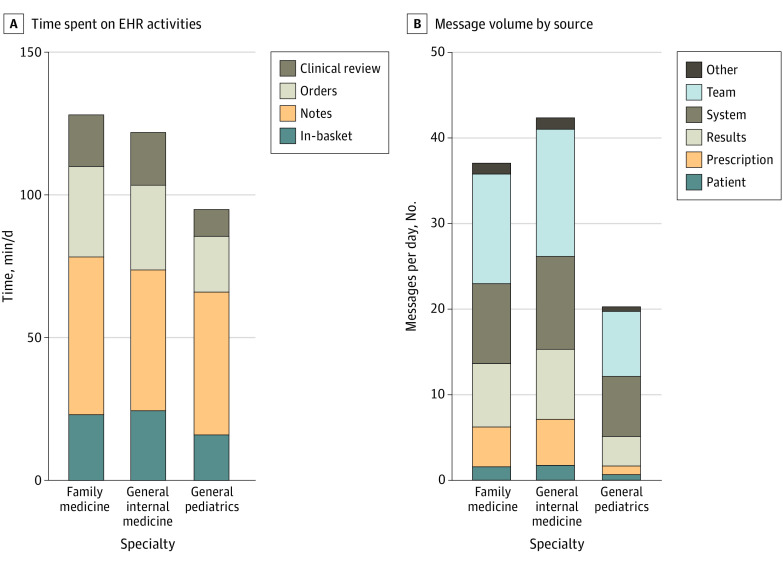
Distribution of Time Spent on Electronic Health Records (EHR) by Primary Care Specialty Type

Pediatric clinicians spent approximately half as long on in-basket messages (mean [SD], 9.4 [4.1] minutes) as family medicine (mean [SD], 18.0 [6.0] minutes) and general medicine (mean [SD], 18.4 [7.2] minutes) clinicians, and two-thirds as much time on clinical review and orders (Figure 2). Time spent on notes was comparable among primary care specialties. Compared with family medicine and general medicine clinicians, pediatric clinicians received one-fifth as many prescription messages, one-third as many patient messages, one-half as many team messages, and less than one-half as many results messages (Figure 2).

## Discussion

This cross-sectional study found that pediatric clinicians spent significantly less total and after-hours time actively using the EHR compared with general medicine and family medicine clinicians, even after adjusting for organizational characteristics and clinical volume. While previous studies have detailed how pediatricians and internists use the EHR,^4,5^ we found significant differences in EHR use across primary care specialties.

Some differences may be attributed to the lesser medical complexity of pediatric patients. It is also possible that pediatricians are spending more time on clinical care outside of the EHR. However, it is notable that time spent on notes was similar across specialties, suggesting that documentation burdens may be driven by factors beyond patient complexity.

Our study’s strengths include the availability of granular EHR metrics and data from a broad set of institutions. Limitations include a measure of EHR time that considers only active use and may differ from other measures used in the field,^6^ use of data from a single EHR vendor, and inability to adjust for panel or clinician characteristics.

Our findings suggest that there was a disproportionate burden of EHR time for general internal medicine and family medicine clinicians compared with pediatric clinicians. While some differences may be influenced by differences in clinical complexity, our findings highlight opportunities to streamline diverse EHR functions, with particular focus on messaging functions, for adult primary care clinicians and to streamline documentation requirements for all primary care clinicians.

## Notes

Supplement. eMethods.
