# Supplementary material for: Differences in Clinician Electronic Health Record Use Across Adult and Pediatric Primary Care Specialties
Source: JAMA Netw Open. 2021 Jul 9;4(7):e2116375. doi: 10.1001/jamanetworkopen.2021.16375 (PMC8271359; doi:10.1001/jamanetworkopen.2021.16375)
Supplement: Supplement. — eMethods. [file jamanetwopen-e2116375-s001.pdf]

Supplemental Online Content

Rotenstein LS, Holmgren AJ, Downing NL, Longhurst CA, Bates DW. Differences in clinician electronic health record use across adult and pediatric primary care specialties. *JAMA Netw Open*. 2021;4(7):e2116375. doi:10.1001/jamanetworkopen.2021.16375

eMethods

This supplemental material has been provided by the authors to give readers additional information about their work.

© 2021 Rotenstein LS et al. *JAMA Network Open.*

eMethods

Measurement of Activity

Epic Systems monitors user activity in the EHR at a granular level, collecting what is commonly referred to as metadata. Metadata document time spent actively using the system indicated by keystrokes, mouse movement, clicks, scrolling, and other interactions within the software. In this analysis, "active time" within an EHR is defined as time a user is performing active tasks. If no activity is detected for 5 seconds, the system stops counting time. This measurement allows our data to capture actual EHR work while excluding time a clinician spends with the EHR open but performing other tasks. This is an inherently conservative measure of EHR time but is available from metadata in a standardized fashion across all study sites. However, this measurement may result in an underestimate of true EHR work time because clinicians often spend time reading notes or otherwise performing EHR tasks without directly interacting with the system. Thus, our EHR active work time should be thought of as a measure related to but distinct from previous studies measuring clinician EHR time by methods such as audit log data or time-and-motion studies.

Definitions of Total and After-Hours Time

Using the definition of active time above, we measured total time actively using the EHR per clinician per day. We additionally calculated the amount of after-hours time clinicians spent working in the EHR each day. We defined after-hours EHR use as any time between the hours of 5:30 P.M and 7:00 A.M. local time on weekdays and any time on weekends, unless the clinician was scheduled during those times. We only included time spent performing work for clinical activities and excluded time spent in the EHR for research, data analysis, performance measurement, or system customization.

© 2021 Rotenstein LS et al. *JAMA Network Open.*
